# Microbial biogeography of a university campus

**DOI:** 10.1186/s40168-015-0135-0

**Published:** 2015-12-01

**Authors:** Ashley A. Ross, Josh D. Neufeld

**Affiliations:** Department of Biology, University of Waterloo, 200 University Avenue West, Waterloo, Ontario N2L 3G1 Canada

**Keywords:** University campus, Door handles, Microbiome, Human skin, High-throughput sequencing, Built environment, Outdoor microbiology, Biofilm

## Abstract

**Background:**

Microorganisms are distributed on surfaces within homes, workplaces, and schools, with the potential to impact human health and disease. University campuses represent a unique opportunity to explore the distribution of microorganisms within built environments because of high human population densities, throughput, and variable building usage. For example, the main campus of the University of Waterloo spans four square kilometres, hosts over 40,000 individuals daily, and is comprised of a variety of buildings, including lecture halls, gyms, restaurants, residences, and a daycare.

**Results:**

Representative left and right entrance door handles from each of the 65 buildings at the University of Waterloo were swabbed at three time points during an academic term in order to determine if microbial community assemblages coincided with building usage and whether these communities are stable temporally. Across all door handles, the dominant phyla were *Proteobacteria*, *Firmicutes*, *Actinobacteria*, and *Bacteroidetes*, which comprised 89.0 % of all reads. A total of 713 genera were observed, 16 of which constituted a minimum of 1 % of the 2,458,094 classified and rarefied reads. Archaea were found in low abundance (~0.03 %) but were present on 42.8 % of the door handles on 96 % of buildings across all time points, indicating that they are ubiquitous at very low levels on door handle surfaces. Although inter-handle variability was high, several individual building entrances harbored distinct microbial communities that were consistent over time. The presence of visible environmental debris on a subset of handles was associated with distinct microbial communities (beta diversity), increased richness (alpha diversity), and higher biomass (adenosine 5′-triphosphate; ATP).

**Conclusions:**

This study demonstrates highly variable microbial communities associated with frequently contacted door handles on a university campus. Nonetheless, the data also revealed several building-specific and temporally stable bacterial and archaeal community patterns, with a potential impact of accumulated debris, a possible result of low human throughput, on detected microbial communities.

**Electronic supplementary material:**

The online version of this article (doi:10.1186/s40168-015-0135-0) contains supplementary material, which is available to authorized users.

## Background

Antonie van Leeuwenhoek first discovered organisms living on surfaces linked to the human body in the seventeenth century using simple microscopes [1]. For the next three centuries, studies of human-associated microorganisms focused on the diversity, morphology, and metabolism of a limited group of cultured isolates. Organisms that were initially cultured from healthy skin in the 1950s include *Staphylococcus epidermidis*, *Micrococcus*, and *Propionibacterium* [2]. With the advent of modern molecular approaches, the microbiome of each region of the human body can now be explored thoroughly.

Skin is the largest organ of the body and is comprised of a diverse range of mostly harmless and beneficial organisms [3]. The skin microbiota varies between body sites and individuals, exhibiting greater collective diversity than both the human oral cavity and gut [4]. In a survey of skin microbiota, *Propionibacteria*, *Corynebacteria*, and *Staphylococcus* spp. comprised over 62 % of sequences detected across 20 body sites [5]. *Corynebacteria* spp. associated with moist skin, *Propionibacteria* and *Staphylococcus* spp. dominated sebaceous areas, and both *Betaproteobacteria* and *Flavobacteriales* were abundant in dry regions. Using 3D molecular cartography maps and a survey of skin microbiota across ~400 human skin sites, a recent study demonstrated that *Actinobacteria*, *Firmicutes*, *Proteobacteria*, *Cyanobacteria*, and *Bacteroidetes* were the most common phyla detected [6]. Previous research demonstrated that the majority of the human skin microbiota are temporally stable [4], yet hand microbial communities are dynamic and possess a larger proportion of transient organisms [7, 8]. Despite the majority of these organisms being benign or beneficial for health, transient and opportunistic pathogens are present that can cause several common human diseases. For example, *Staphylococcus aureus*, *Corynebacterium minutissimum*, and *Pseudomonas aeruginosa* are the respective causes of atopic dermatitis, erythrasma, and green nail syndrome [9].

Human skin is of interest for the study of the built environment because, in addition to possessing its own microbial community, skin commonly represents the first point of contact between humans and microbes. In contrast to previous generations, inhabitants of industrialized countries spend the majority of their time indoors [10], in daily contact with a variety of surfaces at home, work, and when traveling. Studies that examine human-associated built environments are important for better understanding the microbes that humans encounter frequently, which has implications for health and disease. Indeed, recent studies focusing on the built environment found that public restroom surfaces host diverse microbial communities that are primarily composed of human-associated organisms [11]. This suggests that microorganisms on skin are deposited during short periods of contact. This is further supported by the finding that microorganisms deposited on computer keyboards resemble those from the hands that typed on them [12], which has potential forensic implications.

Surface-associated bacterial communities exhibit seasonal variation, although differences in building use have a larger impact on the community than the time of the year [13]. Homes were found to contain a variety of communities whose composition was impacted directly by the usage of the surfaces that were swabbed. Surfaces that were cleaned frequently had a lower diversity than unwashed surfaces [14]. In addition, homes with dogs had higher bacterial diversity and relative abundance of dog-associated bacteria on the majority of surfaces. Interestingly, the microbial community on kitchen surfaces in the home could be linked to the microbial communities associated with the house’s human inhabitants [7]. *Firmicutes*, *Bacteroidetes*, *Actinobacteria*, and *Proteobacteria* were present in the majority of household surface samples, as well as on human skin. When families moved, the new house was associated with a distinct community that reflected the skin community of the new inhabitants [7]. The spread of this distinct community has recently been associated with a “microbial cloud”, where individuals within a room can be detected through airborne microorganisms and settled particles within 1.5–4 h after entering [15]. These results reinforce the importance of occupants in shaping microbial communities associated with building surfaces [16].

University campuses are unique built environments with high population densities and throughput, in addition to being represented by buildings with distinct uses. For example, the University of Waterloo is comprised of 65 buildings within four square kilometers. The main campus has almost 40,000 full-time students and 4000 staff and faculty. Campus buildings are widely varied in their usage, including lecture halls, gyms, health and optometry clinics, dormitories, a day care, and restaurants. A common feature of these buildings is their entranceways. Most campus buildings have metal door handles that are touched by many students each day, presumably exchanging a portion of their personal skin microbiota with contacted surfaces.

The public health importance of these university-based built environment interactions is highlighted by previous work demonstrating that pathogenic bacteria can survive on metal surfaces for extended periods of time, even weeks [17]. Exacerbating the numerous colds and flus that are transmitted in dormitories and lecture halls, some university students may not be diligent hand washers. In an observational study at a large public university in Texas, over 25 % of university students did not wash their hands after each bathroom use [18]. Of students that washed their hands, 58 % used soap and only 26 % employed an adequate hand washing technique. Inadequate personal hygiene can increase the diversity of microorganisms on hands and, by extension, on contacted surfaces [8]. Of particular interest is the finding that hand washing practices varied greatly within a university based on the designated use of the building that a student occupies [18]. Specifically, students were more likely to wash their hands in the bathroom of an academic building than in a recreation center.

The main objective of this study was to determine if university campus buildings host distinct microbial communities on door handle surfaces that reflect building usage. An additional objective was to evaluate whether door handle surfaces were temporally stable throughout an academic term. We show that although door handle communities were highly variable overall, microbial community profiles can be unique to several buildings and, in some cases, remain relatively stable over time. Furthermore, we identified that visible debris on a door handle, which may reflect building throughput, is associated with distinct microbial communities and increased biomass.

## Results and discussion

### Campus door handle communities and human skin

**Fig. 1:**
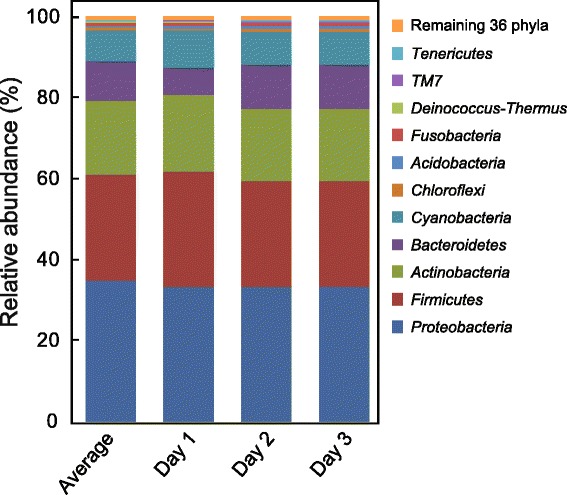
Relative abundance of the 47 phyla found on 65 university campus building door handles. The *Average* data represent pooled sequences from all three time points

We examined alpha and beta diversity of bacteria and archaea on 130 door handles from 65 university buildings over three time points, for a total of 390 samples. Altogether, 12,624 operational taxonomic units (OTUs) were obtained from 2,458,094 reads from 383 samples, after the data were rarefied to 6418 reads per sample. The four dominant phyla were *Proteobacteria*, *Firmicutes*, *Actinobacteria*, and *Bacteroidetes*, which constituted an average of 89.0 % of all reads (Fig. 1). These dominant phyla were also associated with previous built environment studies of public restroom surfaces [11], homes [7], and gym surfaces [19]. The same four phyla are also dominant on human skin [8]. The remaining 11.0 % of all reads affiliated with 43 other bacterial and archaeal groups (Fig. 1). Overall, the proportion of each detected phylum differed by 0.0002–4.35 % across the three time points (Additional file 1: Table S1), indicating that overall door handle community composition remained relatively stable temporally, at least at the phylum level. Included in these additional phyla are a relatively large proportion of sequences affiliated with *Cyanobacteria*, which have previously been found on classroom walls and floors, attributed to soil and bioaerosol accumulation [20]. As expected, the majority of detected cyanobacterial sequences affiliated with chloroplasts (>85 %; Additional file 1: Table S1), which may have been deposited as pollen, plant material, or residual food from hands. In addition, a recent study showed that skin-care products based on plant extracts, used on the hands of a sampled individual, resulted in the detection of cyanobacterial sequences across multiple skin locations of that same individual [6], yet not on the other individual included in that survey.

**Fig. 2:**
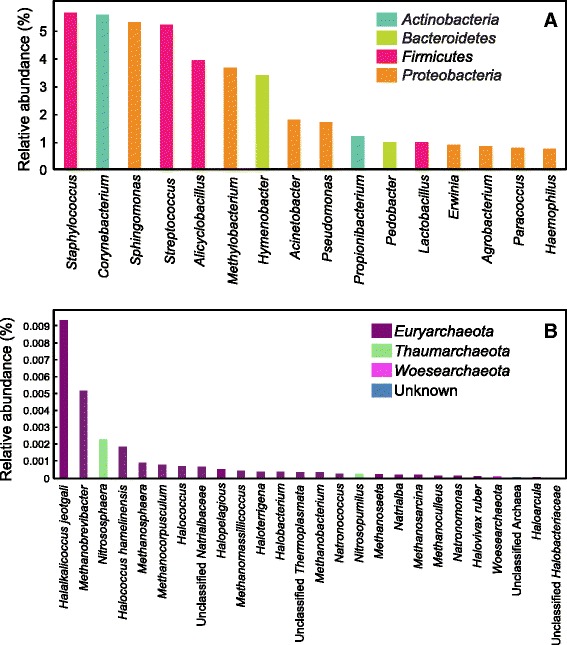
The relative abundance of the most prevalent bacterial and archaeal OTUs. **a** The 16 bacterial OTUs that make up >1 % of all reads and the phylum to which they belong. **b** All 26 archaeal OTUs and their respective phyla

There were 16 OTUs that dominated door handle microbial communities, associated with >1 % of total reads each, together representing 42.4 % of all reads (Fig. 2a). All 16 of these dominant OTUs corresponded to members of one of the main four phyla found on human skin and built environment surfaces (Fig. 1). *S. epidermidis*, *Streptococcus*, *Sphingomonas*, *Alicyclobacillus*, and *Methylobacterium* were found on all 383 door handle samples with sufficient sequences for analysis (Additional file 1: Table S1). *Staphylococcus*, *Streptococcus*, *Corynebacterium*, and *Propionibacterium* have previously been found on airplane surfaces, including door handles, at high abundance [21]. Note that an important limitation of 16S ribosomal RNA (rRNA) genes is that very little evidence for pathogenicity, or lack thereof, can be attributed to detected sequences. Nonetheless, if some of the sequences detected in this study corresponded to opportunistic pathogens, door handle surfaces would be likely to promote their distribution, which is a situation that is potentially exacerbated by lack of handwashing observed previously by university students [18].

Several of the abundant organisms observed in this study have the potential to form a biofilm on door handles (Fig. 2a). Estimates suggest that 75.6 % of 119 tested strains of *Corynebacterium* isolated from human back and forehead skin can form biofilms on artificial solid surfaces [22]. *Staphylococcus*, *Streptococcus*, and *Sphingomonas* have also been found to form biofilm on solid surfaces [23–25]. Similar observations were made with *Acinetobacter*, *Pseudomonas*, *Methylobacterium*, and *Corynebacterium*, which were found together on stainless steel biofilms [26]. We hypothesize that dead skin, oils from the hand, soil, and other organic matter supply sufficient nutrients and moisture for microorganisms to form a stable outdoor entrance handle community.

Because we used universal prokaryotic primers to amplify both archaeal and bacterial DNA, archaea were detected, yet were in very low abundance compared to bacteria, constituting only 0.03 % of all reads. The primer set used matches 94.6 % of archaeal reads when compared to the RDP database [27]. Thus, although archaeal taxa are clearly less abundant than bacteria, a portion of the reduced abundance may be due to primer mismatches. Archaea were found on 164 of 383 door handle samples; 4 of the 65 sampled buildings had no archaeal reads at any time point or entrance. These buildings without detected archaea consisted of an engineering lecture hall and three dormitories. No buildings had archaeal reads on all door handles at all time points.

The dominant archaeal phylum detected was *Euryarchaeota*. Indeed, of the 26 distinct genera, 22 were affiliated with *Euryarchaeota* (Fig. 2b). The majority of the archaeal organisms detected by sequences consisted of halophilic archaea and methanogens. Both halophiles and methanogens have been observed on human skin and commonly in the human gastrointestinal tract [28]. As a result, we hypothesize that the majority of the archaea were deposited from human-associated tissue such as skin and the gastrointestinal tract. Two OTUs belonged to the *Thaumarchaeota* (*Nitrosophaera* and *Nitrosopumilus*), which have been found on human skin and in marine environments [29, 30]. Thaumarchaeotes have also been discovered in engineered freshwater environments, such as wastewater treatment plants [31] and aquaria [32, 33]. The remaining classified genus associated with door handle sequences belonged to newly discovered anaerobic carbon cyclers associated with *Woesearchaeota* [34]. The most abundant archaeal OTU, comprising 35.4 % of all archaeal reads, was *Halalkalicoccus jeotgali*, which is an organism originally isolated from fermented Korean food [35]. Alongside other halophilic archaeal OTUs and the bacteria *Alicyclobacillus* and *Lactobacillus*, these data suggest that deposition of microorganisms from food contributed to OTUs detected on door handles.

### Door handle diversity

**Fig. 3:**
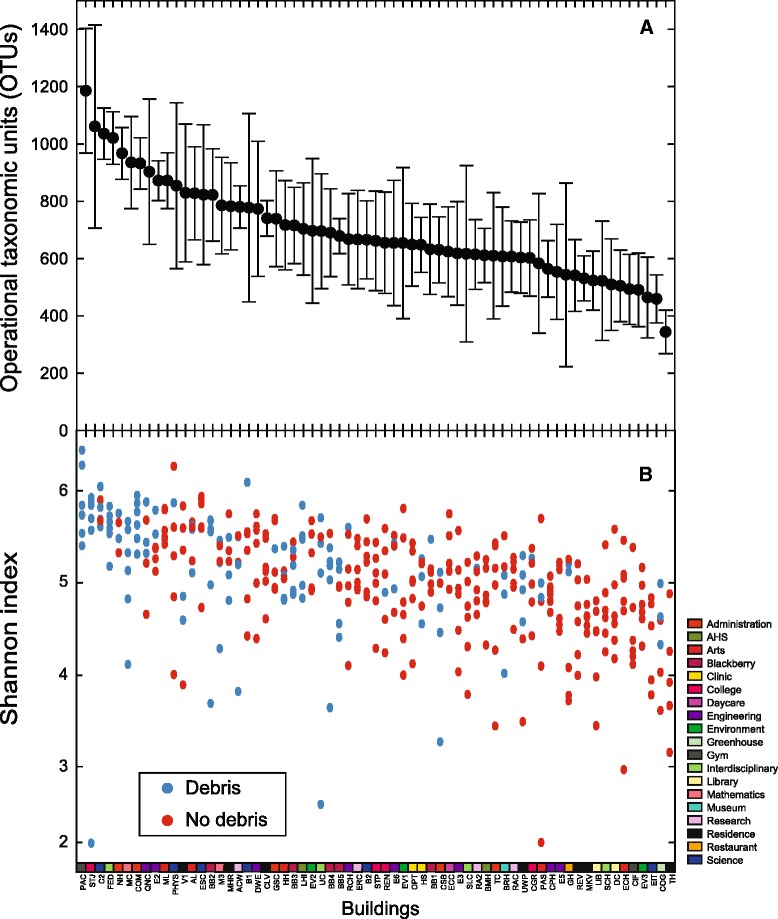
Diversity of the 65 university campus buildings. **a** The average number of OTUs observed for each building. Each point consists of up to six samples, with two per sample date. *Error bars* represent the standard deviation. **b** Shannon index for each door, organized by building. *Blue points* represent swabs that were associated with visible debris, whereas *red points* indicate swabs without debris. The bottom row is color coded according to the faculty or specific campus function of the building

The number of OTUs on door handles ranged from 221 to 1450 (Fig. 3a). The building with the lowest average number of OTUs was the Tutor House (TH) student townhouses, whereas the building with the most OTUs was the Physical Activities Complex (PAC). In particular, the PAC had the most OTUs during time point 2 when a student convocation event was occurring inside. Samples from this time point had an average of 754 more OTUs than all other samples from all sampling dates and an average of 393 more OTUs than PAC door handles from the remaining sampling dates (Additional file 1: Table S1). Because convocation brings thousands of visitors to the university, and the PAC in particular, we predict that increased student throughput and the presence of visitors who do not normally contact door handles on campus provides a reasonable explanation for the increase in diversity observed on the PAC door handles at this time. This finding was reinforced by the adjacent Student Life Centre (SLC) building, which is also used during convocation events. The SLC had an average of 319 more OTUs during convocation than the SLC door handles from the remaining sampling dates and 140 more OTUs than all other door handle samples from all sampling dates.

**Fig. 4:**
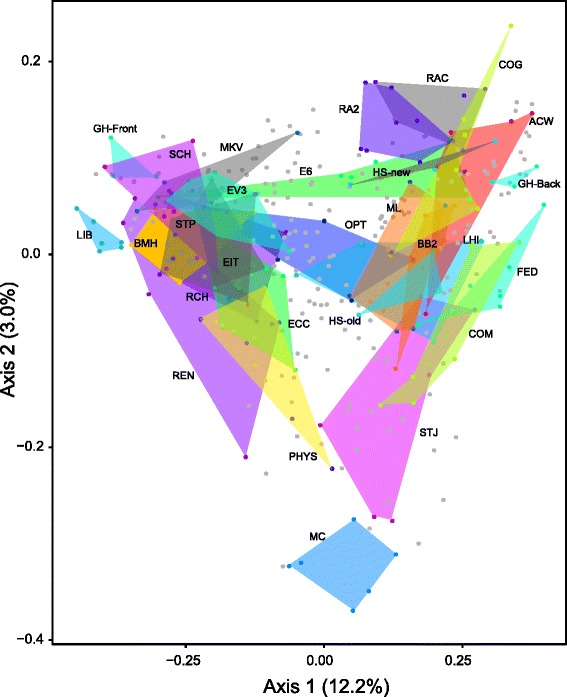
PCoA plot calculated using the Bray-Curtis dissimilarity metric. The 383 samples from all time points are included and organized by building. *Polygons* encompass all samples from 25 buildings that remained relatively stable over the course of the study. Samples from remaining buildings, which were highly variable over time, are shown as *gray points*. Abbreviations are explained in Additional file 3: Table S2

In order to visualize beta diversity, principle coordinates analysis (PCoA) plots were generated based on the Bray-Curtis dissimilarity metric (Fig. 4). Reflecting the high heterogeneity of the sampled door handle surfaces, only 12.2 % of variance was explained by the primary axis and 3.0 % was explained by the secondary axis. Such high heterogeneity was also reflected by a similarly low degree of variation explained by the primary (16.8 %) and secondary (10.9 %) axes associated with a study of gym surface microbial communities [19], which was based on another weighted distance metric (weighted UniFrac). This also correlates with previous findings that communities found within a surface type (such as multiple doors) are more heterogeneous than communities found between different surface types (such as a door and nearby floor) [20, 36]. Together, these studies demonstrate that samples from different sources, such as phones and shoes, generally have more distinct groupings in ordinations than samples from a single source.

The distribution of door handle surfaces within ordination space revealed that, in many cases, individual buildings were akin to microbial “islands”, possessing distinct door handle profiles that persisted temporally (Fig. 4). Supporting this initial observation, and in order to identify links between metadata and detected door handle communities, we used Multi-Response Permutation Procedures (MRPP) as a nonparametric method for testing differences between a priori defined groups [37]. The building category had the largest *A* value of 0.108 and a test statistic (*T*) value of −30.7. All other categories had *A* values <0.1. Many ecology studies consider *A* values >0.1 to be significant and more negative test statistic (*T*) values also indicate a larger effect size [38]. An analysis of similarities (ANOSIM) was also conducted to identify categories that accounted for differences in community composition. An *R* value of 0 suggests even distribution between categories, whereas 1 suggests the groups are dissimilar [39]. Using 1000 permutations, buildings were the main factor influencing differences in microbial communities (*R* = 0.41, *p* < 0.001). There were also distinguishable communities associated with the building usage (*R* = 0.19, *p* < 0.001), the presence of debris (*R* = 0.19, *p* < 0.001), the expected human throughput (*R* = 0.14, *p* < 0.001), and the size of the building (*R* = 0.12, *p* < 0.001). All other categories had *R* values of <0.1, indicating microbial communities detected on samples sorted by these designations were not distinguishable from one another.

### Door handles and visible debris

The presence of debris, defined subjectively as visible residue on the swab surface following sampling, was observed on 123 of 383 samples (32.1 %). The above statistical analyses showed that door handle communities were influenced by the presence of such a visible film on door handles. The Shannon index was 5.20 ± 0.65 for debris-associated swab samples, in comparison to 4.94 ± 0.58 for non debris-associated swabs (Fig. 3b). Door handles with debris had a higher average number of OTUs (815 ± 227) than those without debris (638 ± 204). This represents a statistically significant difference between the two categories (*p* < 0.001).

An indicator species analysis was performed to identify taxa that affiliated with “debris” or “no debris” samples, with a indicator value threshold of 0.7, a median sequence count >10, and *p* < 0.001. The set of no debris indicators reflected human-associated sources (Table 1). For example, *Rothia dentocariosa* is commonly found in the human oral tract [40], whereas *Streptococcus*, *Corynebacterium*, and *Propionibacterium acnes* are abundant on skin [3]. In contrast, the debris indicators *Bacillus* [41] and *Geodermatophilaceae* [42] were characteristic of taxa commonly found in soil. In addition to indicator species, the core organisms that were found in every sample varied if debris was observed (Table 2). Aside from the five organisms found on every door handle, only a single OTU, affiliated with *P. acnes*, was detected on all door handles without debris. In contrast, door handles with debris had 10 OTUs that were core to all samples. Together, these findings demonstrate that the possible presence of soil/dust accumulation within oils deposited on door handles can shift a microbial community away from a skin-associated community typical of the built environment.

**Table 1:** Indicator species for building and debris categories

Category	Category info	Organism	Indicator value	Median sequences
Building	Blackberry 1	*Deinococcus*	0.99	12
		*Hymenobacter*	0.81	10
		*Nocardiaceae*	0.79	200
		*Williamsia*	0.70	354
	Blackberry 2	*Chloroflexi*	0.89	64
		*Frankiaceae*	0.77	49
	St. Jerome’s	*Clostridium bowmanii*	0.73	153
Debris presence	Yes	*Bacillales*	0.70	14
		*Geodermatophilaceae*	0.70	14
	No	*Corynebacterium*	0.73	51
		*Rothia dentocariosa*	0.71	11
		*Streptococcus*	0.71	236
		*Propionibacterium acnes*	0.71	74

All *p* values <0.001. Included significant indicators with indicator value >0.7 and median number of sequences >10. No other buildings had indicator species that met the threshold

**Table 2:** Phylogenetic affiliations of core taxa on door handles with and without debris

No debris	Debris
*Propionibacterium acnes*	*Erwinia*
	*Comamonadaceae*
	*Hymenobacter*
	*Nocardiadaceae*
	*Mircobacterium*
	*Paracoccus*
	*Geodermatophiliceae*
	*Oxalobacteriaceae*
	*Pseudomonas viridiflava*
	*Salinibacterium*

**Fig. 5:**
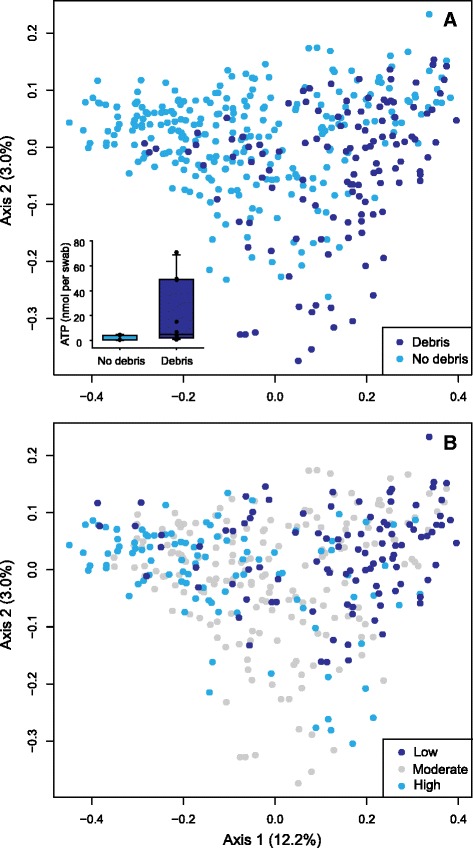
PCoA plot calculated using the Bray-Curtis dissimilarity metric. The 383 samples from all time points are included and organized by debris (**a**) and throughput (**b**). Inset contains the quantity of ATP detected on swabs from 10 university campus door handles that were covered in visible debris and 10 without debris

The presence of debris was also associated with low predicted human throughput for each entrance (Fig. 5). For example, although classified as a low-throughput building, the Grad House is a campus pub that has a front door (GH-front) with relatively higher throughput than the back door (GH-back). With respect to their microbiota, both the front and back Grad House door handles were temporally stable yet distinct from one another within ordination space (Fig. 4). The GH-front door samples associated with other low-debris door handle profiles, whereas the GH-back samples grouped with debris-associated sample profiles (Fig. 5). One explanation is that door handles that are contacted less frequently accumulate dust and wind-distributed soil particles, and the microbes that they carry, within oils that are infrequently deposited on these door handle surfaces. Frequent opening of doors, especially in front of large lecture halls, may prevent the accumulation of debris by physical removal and therefore possess a community that more closely reflects human skin.

To test whether debris-associated door handles were associated with increased viable biomass, we used a bioluminescence assay to measure adenosine 5′-triphosphate (ATP) on door handle surfaces associated with the two category types (Fig. 5a inset; Additional file 2: Figure S1). Although characterized by high variability, door handles with debris had a significantly higher ATP level of 20.13 ± 26.14 nmol per swab compared to the measured ATP levels of 1.71 ± 1.93 nmol per swab on tested door handles without debris (*p* = 0.027). These results suggest that low-throughput debris-associated door handles have more viable organisms than high-throughput handles without visible accumulated debris. To our knowledge, this is the first observation of unique debris-associated communities on built environment surfaces. Further work would be required to better characterize and quantify the content of the debris-associated films detected on specific door handles sampled in this study.

## Conclusions

This study is the first to determine the distribution of bacteria and archaea on entrance door handles of a university campus. Door handles represent built environment surfaces that promote the accumulation and distribution of microbes between individuals. Detected door handle microorganisms were associated with human skin, although the outside entrances were also likely affected by soil, plant material, and food sources. Furthermore, the most substantial influence on the door handle community was the building sampled, such that although the position of buildings in relation to other buildings was insignificant, several buildings housed distinct door handle communities that persisted temporally. Moreover, visible debris that accumulated on door handles with low human throughput were associated with unique microbial communities. Indeed, debris-covered door handles were distinct in microbial composition, more diverse, and contained more viable biomass (ATP) than door handles without visible debris.

Because university campus door handles represent a bottleneck of microbial contact for thousands of students daily, contact with inanimate surfaces represents an important potential mechanism for disease transmission. As a result, understanding factors that influence the accumulation of microbes on door handles provides important baseline data for subsequent studies that might explore mechanisms of pathogen dispersal via built environment surfaces or alternative infrastructure designs that might reduce the potential for microbial accumulation and dispersal.

## Methods

### Sampling

All 65 buildings located at the main campus of the University of Waterloo were sampled. Handles from the left and right doors from the same entranceway of each building were sampled at three time points in 2014: October 17, October 24, and November 7. Where left and right doors were unavailable, multiple doors from the same building were sampled instead (e.g., GH-Front and GH-Back). Each sample was classified by building, faculty, building use, proximity to construction, entrance cardinal direction, left vs. right door handle in a double door entrance, estimated human throughput, nonstandard door handle size, presence of visible debris/deposit on swab, latitude, longitude, building age, number of rooms, building size (net m^2^), and seating capacity (Additional file 3: Table S2). Outside metal door handles were sampled using dry Sterile Foam Tipped Applicators (Puritan). The top, middle, and bottom portions of each door handle were swabbed for 45 s to ensure the entire surface area of the handle was sampled. Swabs were returned to the manufacturer’s individually sealed plastic transport tubes and stored on ice packs in a cooler for transport to the lab, within 3 h, where they were then stored at −20 °C.

### DNA extraction and amplification

Genomic DNA was extracted from the swabs using the PowerSoil-htp 96 Well DNA Isolation Kit (MO BIO Laboratories Inc), following a protocol published previously [8]. In brief, the swab tips were cut with ethanol-sterilized scissors and placed into a well of the bead plate with 750 μL of bead solution. Bead plates were preincubated in a 65 °C water bath for 10 min before proceeding with extraction and purification according to the manufacturer’s protocol. The DNA was eluted in a final volume of 75 μL and stored at −20 °C.

The V3-V4 regions of the 16S ribosomal RNA gene (16S rRNA gene) were amplified by PCR with the universal prokaryotic primers, Pro341Fi and Pro805Ri [27]. These primers amplify both bacterial and archaeal DNA. Each PCR amplification mix contained 2.5 μL of 10× buffer, 1.5 μL of 10 mg mL^−1^ BSA, 0.05 μL of 100 mM dNTPs, 0.05 μL of each 100 μM forward and reverse primers, 0.125 μL of 5 U μL^−1^*Taq* polymerase, 5 μL of template DNA, and deionized water to 25 μL. Each primer pair had an Illumina-specific adaptor and unique barcode integrated into the end of the primer, as described previously [43], allowing all samples to be pooled into a single sample for sequencing. Two sterile swab controls and five no template negative controls were included to monitor potential DNA contamination. All PCR mixes were prepared in a PCR hood that was UV-treated for 15 min. The reaction was 95 °C for 30 s and 35 cycles of 95 °C for 15 s, 55 °C for 30 s, 68 °C for 60 s, and a final extension of 68 °C for 10 min. Each PCR was run in triplicate and then pooled to help minimize amplification bias [44]. PCR products were visualized on 1 % agarose gels with ethidium bromide using standard gel electrophoresis. For all gels, 100 ng of the 1 Kb Plus DNA Ladder (Invitrogen) was loaded alongside samples (5 μL each).

### Sequencing

Pooled amplicons were quantified on a 1.5 % agarose gel containing GelRed (Biotium). The AlphaView Software (Protein Simple) was used to quantify relative intensities of bands. Each plate was then pooled into a single tube by transferring an equal amount of DNA per sample. Pools were purified using the Wizard SV Gel and PCR Clean-up System (Promega) according to the manufacturer’s centrifugation protocol. The final 4-nM pool was quantified using qPCR PerfeCTa NGS Library Quantification Kit for Illumina Sequencing Platforms (Quanta Biosciences), gel quantification, Nanodrop 2000 (Thermo Scientific), and Qubit (Life Technologies). All quantification procedures were followed according to the manufacturers’ protocols.

The amplicon pool was diluted to 10 pM and merged with 5 % PhiX according to the Preparing Libraries for Sequencing on the MiSeq protocol (Part # 15039740 Rev. C; Illumina). High-throughput sequencing on a MiSeq (Illumina) was used for analyzing the amplified 16S rRNA gene V3-V4 region, which resulted in a cluster density of 1073 thousand clusters/mm^2^.

### Sequence analysis

Paired-end sequences were assembled with PANDAseq v. 2.8 [45], analyzed by Quantitative Insights Into Microbial Ecology v. 1.9.0 (QIIME) [46], and managed by automated exploration of microbial diversity v. 1.5 (AXIOME) [47]. The sequences were clustered with UPARSE [48] at 97 % identity and aligned with PyNAST [49]. These open-source software packages were used to assess microbial communities through the generation of phylogenetic trees, identification of OTUs, and downstream statistical analysis [46]. Taxonomy was assigned using the Greengenes training set release 13_8 [50]. The samples were then rarefied to 6418 reads per sample. Of the total 390 samples, 7 were excluded from the study because they did not possess 6418 reads. The eliminated samples were both Needles Hall samples from time point 1, the left handle of Brubacher’s House and the right handles of the Environmental and Information Technology and Tutor’s House doors from time point 2, and the right handle of the Lyle S. Hallman Institute and left handle of the University Club doors, both from time point 3.

Subsequently, both the α- and ß-diversities of the samples were assessed with the remaining 383 samples. In order to measure beta diversity, principle coordinate analysis (PCoA) ordinations were generated based on the Bray-Curtis distance matrix. Sample alpha diversity was measured using the Shannon index. The ANOSIM statistical analysis, using 1000 permutations, was performed using Primer 7 with the PERMANOVA+ add on (PRIMER-E Ltd). Indicator species were classified as those with an indicator value >0.7, a median read count >10, and *p* < 0.001. Core species were classified as those with at least one sequence in the rarefied dataset for each tested category.

### ATP assay

The quantity of ATP on door handles, with and without visible debris, was measured using the ATP Bioluminescent Assay Kit (Sigma-Aldrich), which was modified from a previous protocol [51]. Ten door handles with debris (CSB-R, BB5-R, UC-R, M3-R, C2-R, MC-R, STJ-R, PAC-R, COM-R, FED-R) and ten door handles without debris (BB4-R, ERC-R, QNC-R, BMH-R, B2-R, SLC-R, B1-R, DC-R, ESC-R, GSC-R) were sampled in September 2015. Only one representative door was sampled per building. Each selected door had previously been included in the main sequencing experiment. Debris-positive door handles were initially chosen based on *a priori* knowledge of which buildings typically possessed door handle debris and were confirmed by visually inspecting the swab after sampling. The swab tips were cut with ethanol-sterilized scissors, placed in 1.5-mL centrifuge tubes (Fisherbrand), and covered in 1 mL of boiling MilliQ water. The samples were vortexed for 10 s, cooled on ice, and centrifuged at 10,000×*g* for 30 s. The ATP assay mix (50 μL) was diluted 1:25 in ATP assay mix dilution buffer and added to each well in a white 96-well plate (Costar). Background light emission was measured using the luminescence setting on a FilterMax F5 Multi-Mode Microplate Reader (Molecular Techniques). Supernatant (25 μL), or standard, was added and mixed. A standard curve was created by diluting the ATP standards to a range between 2 × 10^−3^ and 20 μM ATP. The 0.2 μM ATP standard was used to spike each sample for a third luminescence reading to verify that low measured ATP concentrations were not a result of inhibition. All technical measurements were performed in triplicate.

## Availability of supporting data

The sequence data associated with this article are available in the EBI under project accession number PRJEB10962.

## Additional files

Additional file 1: Table S1. Rarefied OTU table organized in multiple tabs. Tab 1 contains the OTU table organized by building, tab 2 contains the identical table organized by sampling day, tab 3 is a condensed table containing only archaeal reads, and tabs 4 and 5 separate the OTU table into samples with and without debris, respectively. Complete sample name codes are explained in Additional file 3: Table S2. A = day 1, B = day 2, C = day 3, L = left door, R = right door, and numbers 1–65 signify each building arranged by consecutive sampling order. Additional file 2: Figure S1. Swabs used in the ATP Bioluminescence Assay, illustrating level of debris swabbed from entrance door handle. Units are in nmol of ATP per swab ± standard deviation of technical triplicates and building codes are also indicated for each swab. Additional file 3: Table S2. Metadata file containing all sample codes, PCR setup information, and sample metadata.
