# Supplementary figures and images for: Microbial biogeography of a university campus
Source: Microbiome. 2015 Dec 1;3:66. doi: 10.1186/s40168-015-0135-0 (PMC4666157; doi:10.1186/s40168-015-0135-0)

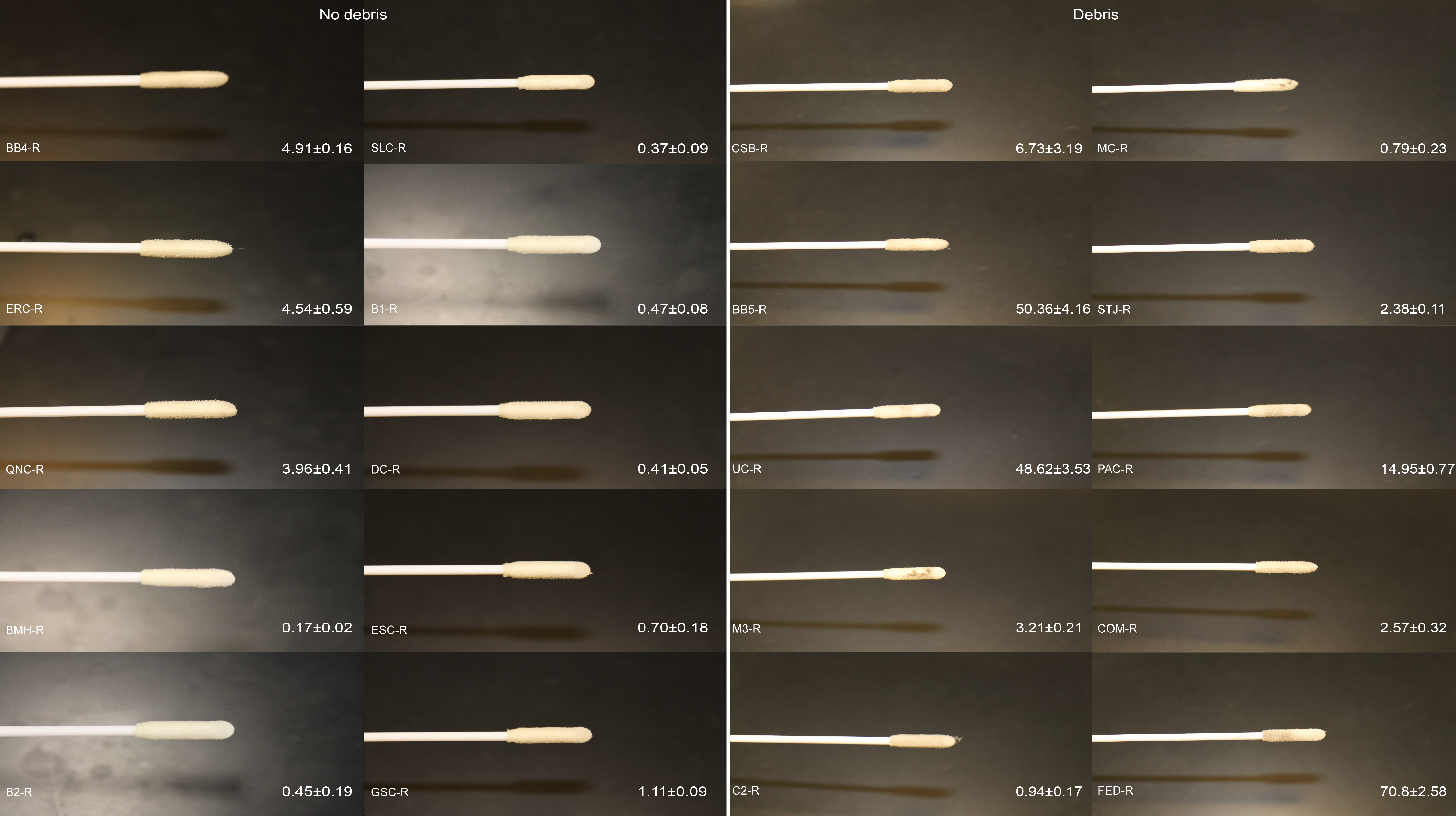

Supplement: Additional file 2: Figure S1. — Swabs used in the ATP Bioluminescence Assay, illustrating level of debris swabbed from entrance door handle. Units are in nmol of ATP per swab ± standard deviation of technical triplicates and building codes are also indicated for each swab. [file 40168_2015_135_MOESM2_ESM.png]
